# Supplementary material for: Population genomics of Group B Streptococcus reveals the genetics of neonatal disease onset and meningeal invasion
Source: Nat Commun. 2022 Jul 21;13:4215. doi: 10.1038/s41467-022-31858-4 (PMC9304382; doi:10.1038/s41467-022-31858-4)
Supplement: Supplementary file 1 — Supplementary information [file 41467_2022_31858_MOESM1_ESM.pdf]

Population genomics of Group B Streptococcus reveals the genetics of neonatal disease onset and meningeal invasion

Chrispin Chaguza, Dorota M. Jamrozy, Merijn W. Bijlsma, Taco W. Kuijpers, Diederik van de Beek, Arie van der Ende, and Stephen D. Bentley

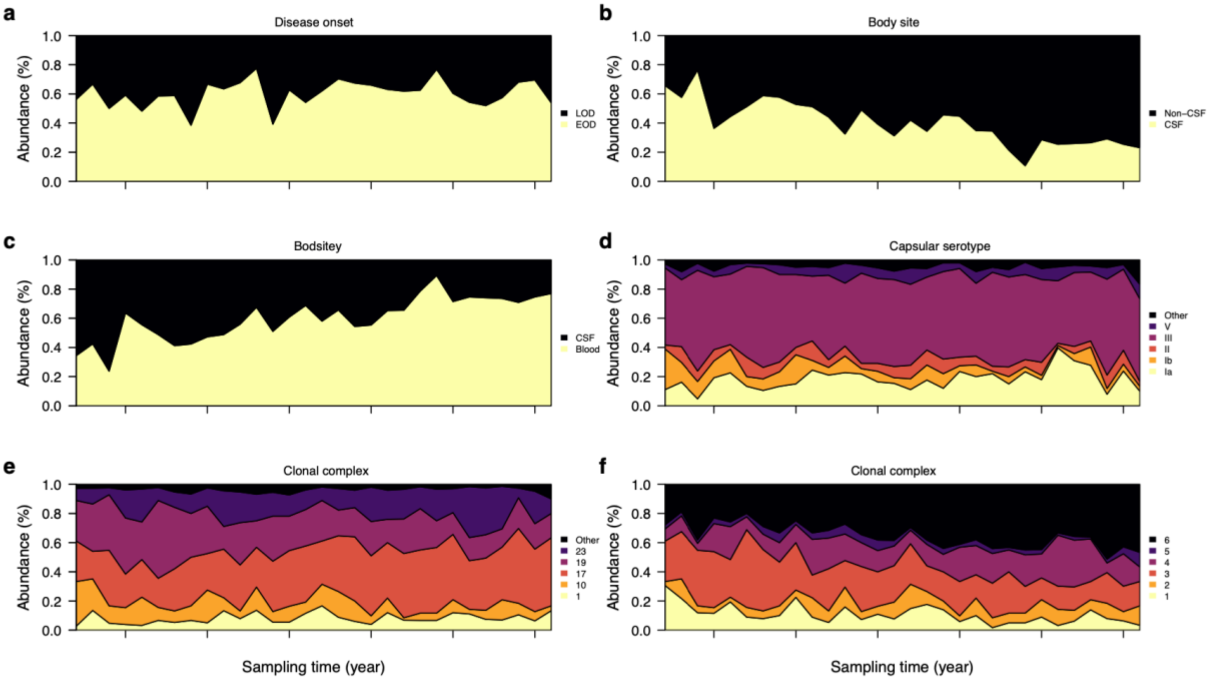

Supplementary Fig. 1. **Annual dynamics of the GBS strains from 1987 to 2016 in the Netherlands. a)** Relative abundance or frequency of the isolates by disease onset, **b)** relative abundance of the isolates by isolation from the central nervous system (CNS) tissue, **c)** relative abundance of the isolates by the body isolation source, **d)** relative abundance of the isolates by capsular serotype, **e)** relative abundance of the isolates by the MLST clonal complex, and **f**) relative abundance of the isolates by the BAPS clade or lineage.

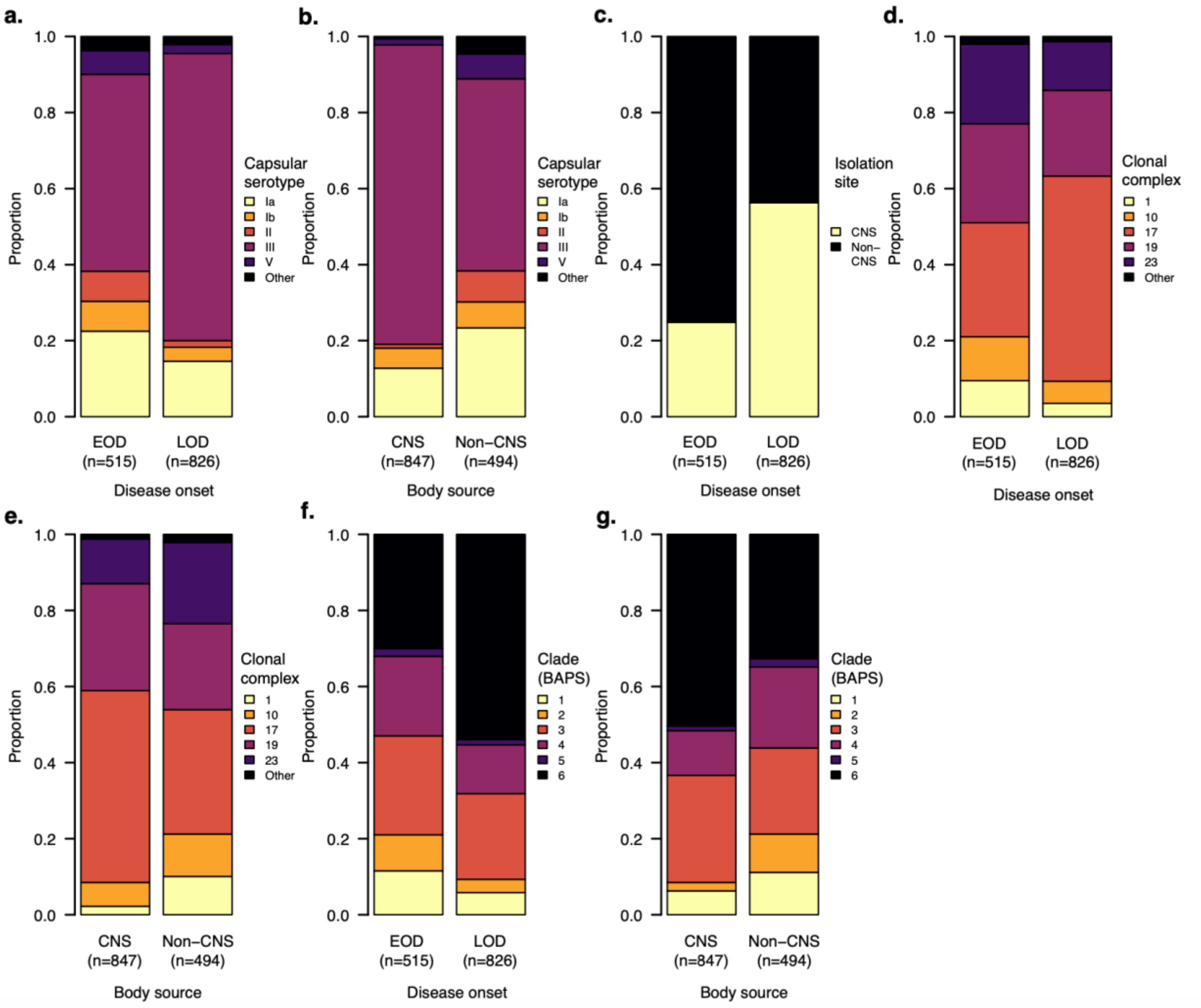

Supplementary Fig. 2. **Overall relative abundance of the GBS isolates between 1987 to 2016 in the Netherlands stratified by disease onset time and CNS infection status. a)** Relative abundance or frequency of capsular serotype by disease onset time, **b)** relative abundance of serotypes by CNS infection status, **c)** relative abundance of the CNS and non-CNS by the disease onset time, **d)** relative abundance of MLST clonal complexes by disease onset time, **e)** relative abundance of capsular serotype by the CNS infection status, **f)** relative abundance of the capsular serotypes by the disease onset time, and **g)** relative abundance of the BAPS clades by the CNS infection status.

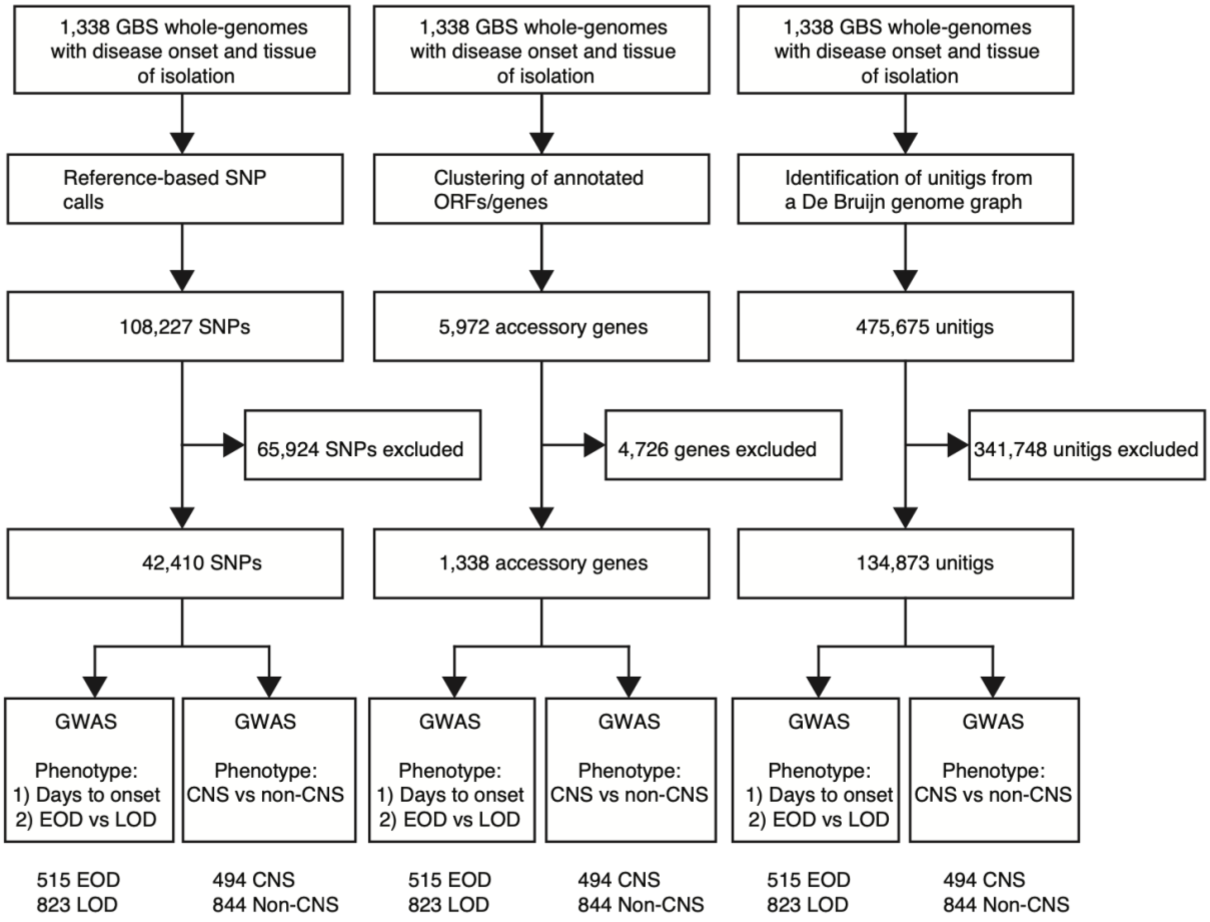

Supplementary Fig. 3. **Flow chart sumarising the number of isolates, phenotypes and genetic variants used in the GWAS analyses.**

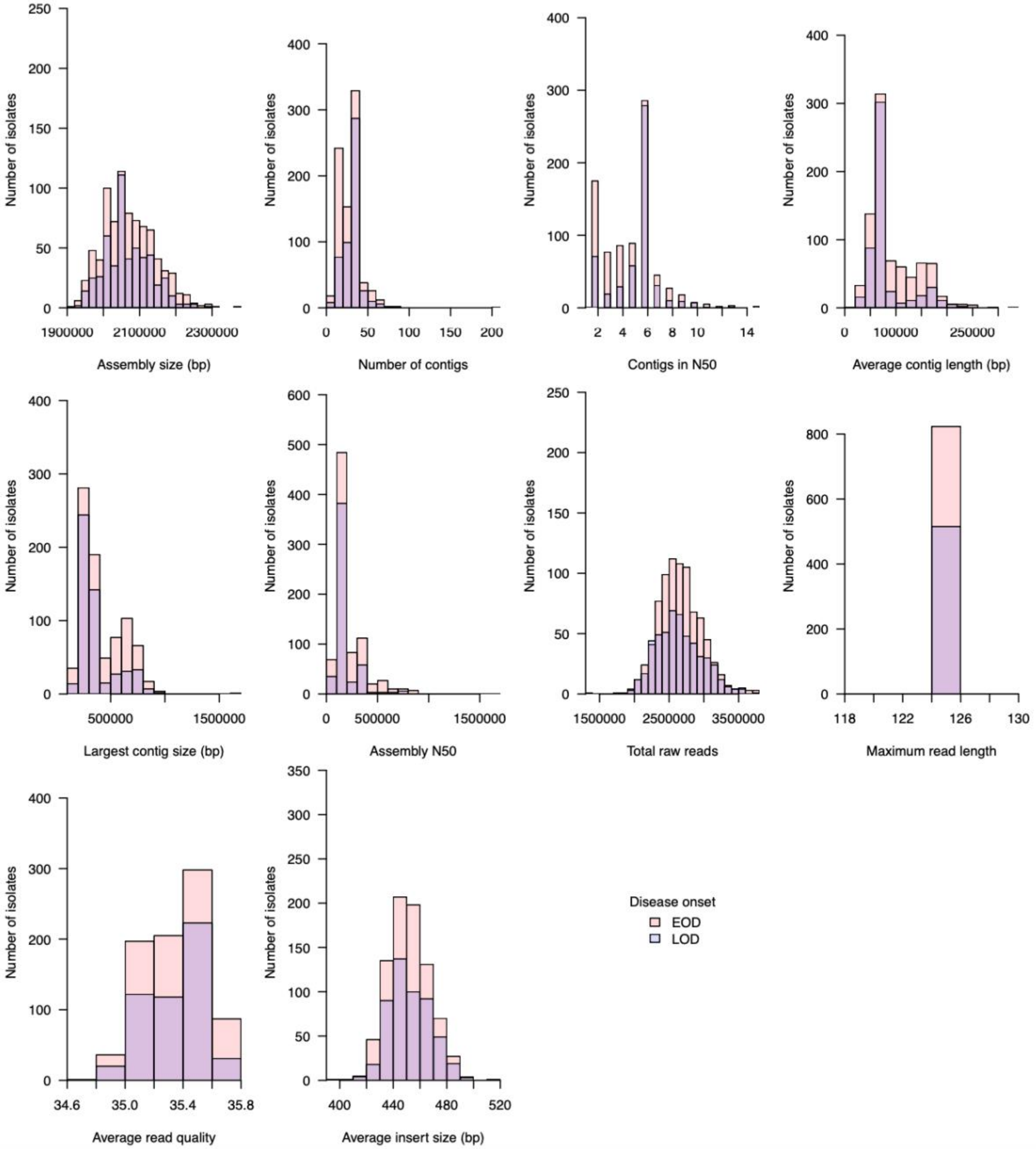

Supplementary Fig. 4. **Summary of the sequencing reads and assembled genomes for the EOD and LOD isolates.**

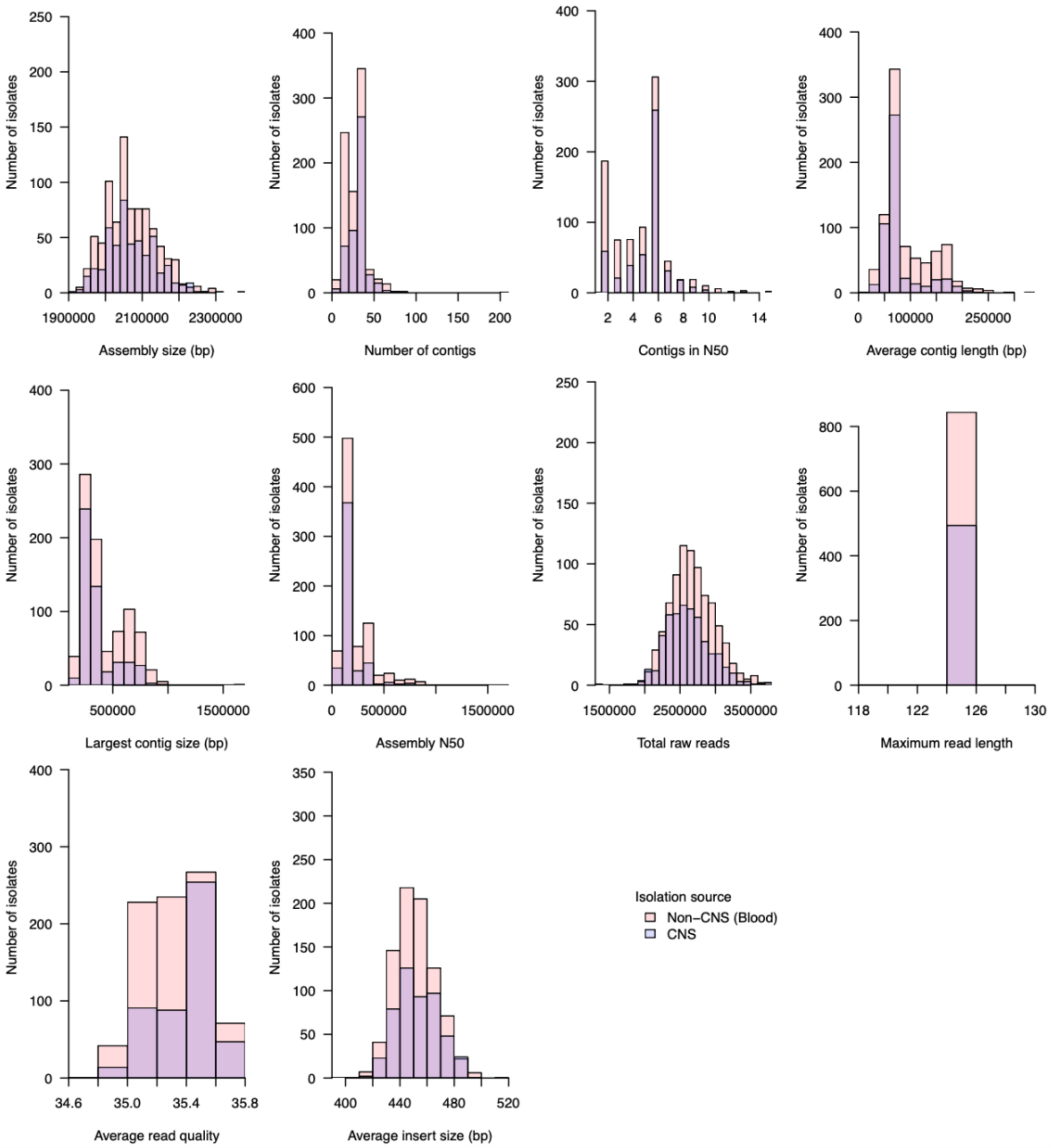

Supplementary Fig. 5. **Summary of the sequencing reads and assembled genomes for the CNS and non-CNS isolates.**

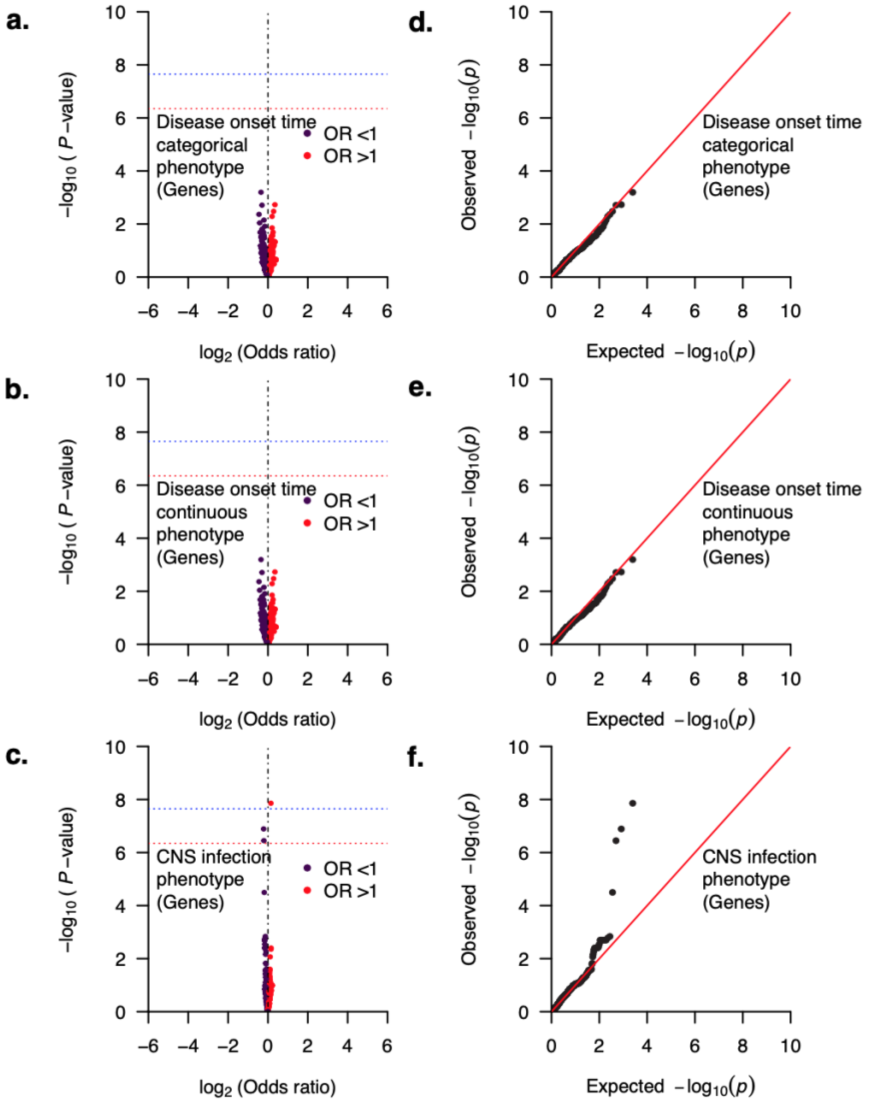

Supplementary Fig. 6. **Summary of the accessory gene-based GWAS using FaST-LMM. (a)** Volcano plot summarising the statistical significance ($-log_{10}P$) based on the likelihood ratio test and effect size ($\log_2$Odds ratio) for the GWAS based on the categorical disease onset time phenotype. **(b)** Volcano plot summarising the statistical significance and effect size for the GWAS based on the continuous transformed disease onset time phenotype. **(c)** Volcano plot summarising the statistical significance and effect size for the GWAS based on the CNS infection phenotype. **(d)** Q-Q plot comparing the observed and expected statistical significance for the GWAS based on the categorical disease onset time phenotype. **(e)** Q-Q plot comparing the observed and expected statistical significance for the GWAS based on the continuous transformed disease onset time phenotype. **(f)** Q-Q plot comparing the observed and expected statistical significance for the GWAS based on the CNS infection phenotype. The red and blue dotted lines represent the genome-wide significance and suggestive threshold, respectively. The variants with odds ratios (OR) >1 is coloured in red while those with odds ratio <1 is coloured in dark purple.

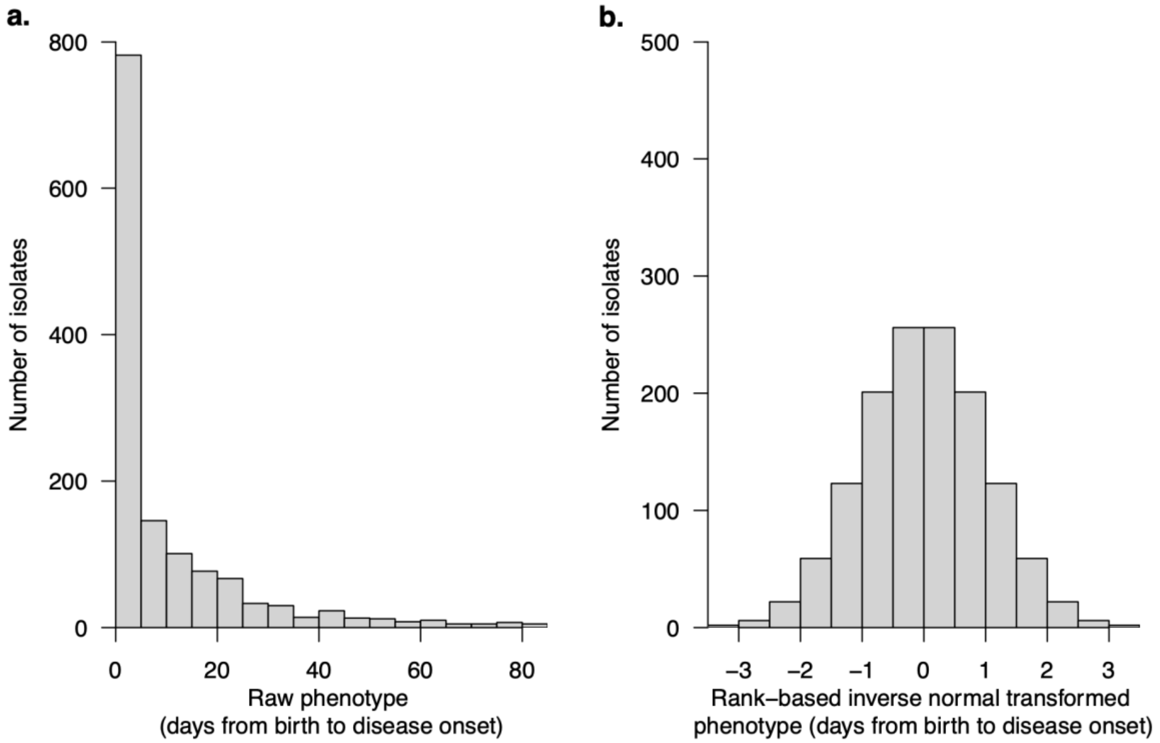

Supplementary Fig. 7. **Distribution of the GBS isolates by the time of acute invasive disease onset. (a)** Untransformed number of days from birth to GBS disease onset, **(b)** rank-based inverse normal transformed number of days from birth to GBS disease onset.

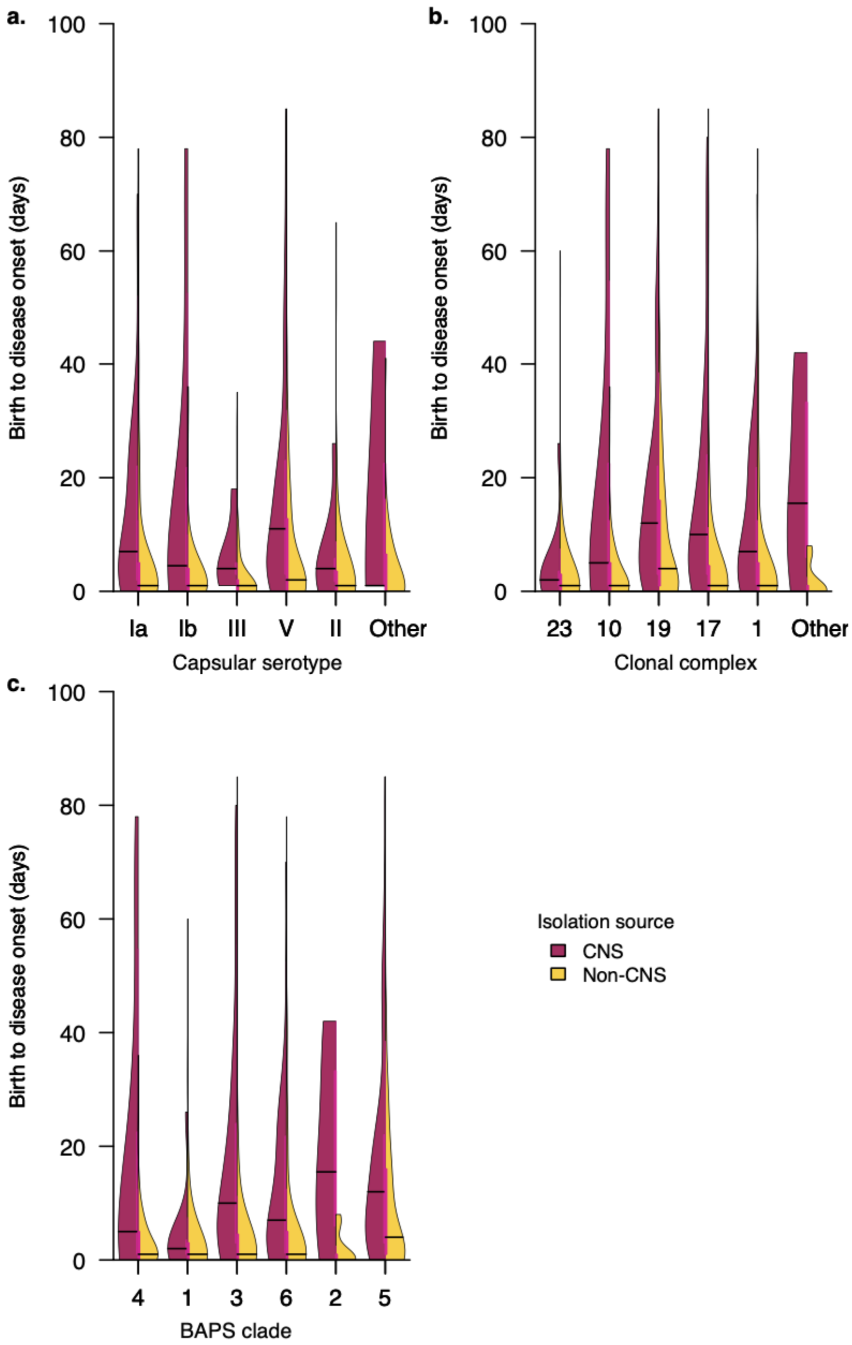

Supplementary Fig. 8. **Distribution of the GBS isolates by the time of acute invasive disease onset.** The Violin plots are stratified by the **(a)** capsular serotype, **(b)** clonal complex, and **(c)** BAPS clades or lineages.

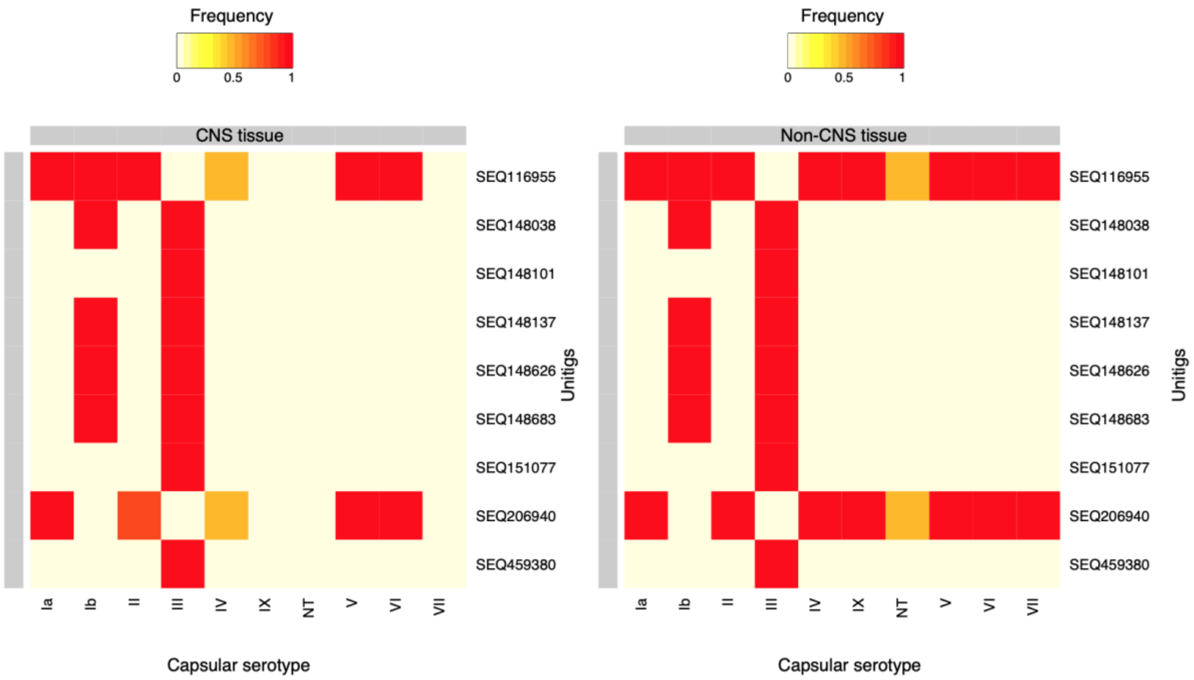

Supplementary Fig. 9. Heatmap showing the relative frequency of unitigs associated with the CNS infection status in isolates of different capsular types
