# Supplementary material for: Population genomics of Group B Streptococcus reveals the genetics of neonatal disease onset and meningeal invasion
Source: Nat Commun. 2022 Jul 21;13:4215. doi: 10.1038/s41467-022-31858-4 (PMC9304382; doi:10.1038/s41467-022-31858-4)
Supplement: Supplementary file 2 — Reporting Summary [file 41467_2022_31858_MOESM2_ESM.pdf]

nature portfolio

Corresponding author(s): Chrispin Chaguza

Last updated by author(s): Jul 5, 2022

Reporting Summary

Nature Portfolio wishes to improve the reproducibility of the work that we publish. This form provides structure for consistency and transparency in reporting. For further information on Nature Portfolio policies, see our Editorial Policies and the Editorial Policy Checklist.

Statistics

For all statistical analyses, confirm that the following items are present in the figure legend, table legend, main text, or Methods section.

n/a	Confirmed	
☐	☒	The exact sample size (*n*) for each experimental group/condition, given as a discrete number and unit of measurement
☐	☒	A statement on whether measurements were taken from distinct samples or whether the same sample was measured repeatedly
☐	☒	The statistical test(s) used AND whether they are one- or two-sided<br>*Only common tests should be described solely by name; describe more complex techniques in the Methods section.*
☐	☒	A description of all covariates tested
☐	☒	A description of any assumptions or corrections, such as tests of normality and adjustment for multiple comparisons
☐	☒	A full description of the statistical parameters including central tendency (e.g. means) or other basic estimates (e.g. regression coefficient) AND variation (e.g. standard deviation) or associated estimates of uncertainty (e.g. confidence intervals)
☐	☒	For null hypothesis testing, the test statistic (e.g. *F*, *t*, *r*) with confidence intervals, effect sizes, degrees of freedom and *P* value noted<br>*Give P values as exact values whenever suitable.*
☒	☐	For Bayesian analysis, information on the choice of priors and Markov chain Monte Carlo settings
☒	☐	For hierarchical and complex designs, identification of the appropriate level for tests and full reporting of outcomes
☒	☐	Estimates of effect sizes (e.g. Cohen's *d*, Pearson's *r*), indicating how they were calculated

Our web collection on statistics for biologists contains articles on many of the points above.

Software and code

Policy information about availability of computer code

Data collection	No software was used for data collection. All the samples used in this study were collected by trained clinicians and processed by an experienced molecular microbiology team. The extracted DNA underwent whole genome sequencing at the Wellcome Sanger Institute, and the resulting data was analysed using the open-source tools listed below.

Data analysis	All software used in the analysis are freely and publicly available. Sequence typing using the multilocus sequence typing (MLST) scheme (https://pubmlst.org/organisms/streptococcus-agalactiae) using SRST2 (version 0.2.0) [https://github.com/katholt/srst2]. Genome assembly was done using SPAdes genome assembler (version 3.14.0) [https://github.com/ablab/spades]. Genomic positions with SNPs were identified using SNP-sites (version 2.3.2) [https://github.com/sanger-pathogens/snp-sites]. Whole genome multiple sequence alignment was generated using Snippy (version 4.6.0) [https://github.com/tseemann/snippy]. Clades or lineages were inferred using BAPS (version 6) [http://web.abo.fi/fak/mnf/mate/jc/software/]. Phylogenetic trees were generated using FastTree (version 2.1.10) [http://www.microbesonline.org/fasttree/]. Phylogenetic trees were visualised and processed using APE package (version 4.3) [https://cran.r-project.org/web/packages/ape/index.html], phylosignal (version 1.3) [https://cran.r-project.org/web/packages/phylosignal/index.html] and phylobase (version 0.8.6) [https://cran.r-project.org/package=phylobase]. Input data for the genome-wide association study (GWAS) were generated using VCFtools (version 0.1.16) [https://vcftools.github.io/index.html]. GWAS files were pre-processed using PLINK (version 1.90b4) [https://zzz.bwh.harvard.edu/plink/index.shtml]. Coding sequences were predicted using Prokka (version 1.11) [https://github.com/tseemann/prokka]. Pangenome analysis was done using Panaroo (version 1.2.2) [https://github.com/gtonkinhill/panaroo]. Unitigs were generated using using Bifrost (version 1.0.1) [https://github.com/pmelsted/bifrost]. GWAS was performed using FaST-LMM (FastLmmC, version 2.07.20140723) [https://github.com/fastlmm/FaST-LMM] and GEMMA (version 0.98.1) [https://github.com/genetics-statistics/GEMMA]. QQ-plots for the GWAS output were plotted using qqman (version 0.1.7) [https://cran.r-project.org/web/packages/qqman/index.html]. Narrow-sense heritability was estimated using FaST-LMM (FastLmmC, version 2.07.20140723) [https://github.com/fastlmm/FaST-LMM], GEMMA (version 0.98.1) [https://github.com/genetics-statistics/GEMMA], and GCTA (version 1.93.2) [https://github.com/jianyangqt/gcta]. Sequence similarity matches were done using BLASTN (version 2.5.0+) [https://blast.ncbi.nlm.nih.gov/Blast.cgi?PAGE_TYPE=BlastDocs&DOC_TYPE=Download]. Genomic sequencing data was processed using BioPython (version 1.78) [https://github.com/biopython/biopython]. Continuous phenotype transformation (https://github.com/ChrispinChaguza/GBS_Study_NL). Results were summarised and plotted using R (version 4.0.3) [https://www.R-project.org/].

For manuscripts utilizing custom algorithms or software that are central to the research but not yet described in published literature, software must be made available to editors and reviewers. We strongly encourage code deposition in a community repository (e.g. GitHub). See the Nature Portfolio guidelines for submitting code & software for further information.

Data

Policy information about availability of data

All manuscripts must include a data availability statement. This statement should provide the following information, where applicable:

- Accession codes, unique identifiers, or web links for publicly available datasets
- A description of any restrictions on data availability
- For clinical datasets or third party data, please ensure that the statement adheres to our policy

The sequence reads for the isolates used in this study are available in the European Nucleotide Archive under study accession code PRJEB14124 (https://www.ebi.ac.uk/ena/browser/view/PRJEB14124). The accession numbers and information for individual isolates are provided in Supplementary Data 1. The authors declare that all other data supporting the findings of this study are available within the paper and its supplementary information files. Additional data for the SNPs, accessory genes, and unitig sequences used in this study are available at https://github.com/ChrispinChaguza/GBS_Study_NL.

Field-specific reporting

Please select the one below that is the best fit for your research. If you are not sure, read the appropriate sections before making your selection.

☒ Life sciences ☐ Behavioural & social sciences ☐ Ecological, evolutionary & environmental sciences

For a reference copy of the document with all sections, see nature.com/documents/nr-reporting-summary-flat.pdf

Life sciences study design

All studies must disclose on these points even when the disclosure is negative.

Sample size	One thousand and three hundred and thirty-eight (1338) GBS isolates were selected for whole genome sequencing from a dataset of isolates collected from nationwide surveillance of infants with bacterial meningitis and bacteraemia conducted by the Netherlands Reference Laboratory for Bacterial Meningitis (NRLBM). No specific sample size calculation was done for this study. In total 823 and 515 isolates were from neonates with early and late onset disease. Based on other bacterial GWAS studies, our study used a larger and more balanced dataset than other studies.
Data exclusions	All the randomly selected bloodstream and cerebrospinal fluid Group B Streptococcus sequenced in this study were included in the analysis. Five isolates sampled from other non-invasive body sites were excluded as the study focused on comparing bloodstream and CSF isolates.
Replication	Replication was not done for this study. Comparable datasets collected annually for a similar period (30 years) are not available.
Randomization	No randomisation was done. We sequenced the available invasive GBS isolates collected from nationwide surveillance of infants with bacterial meningitis and bacteraemia conducted by the Netherlands Reference Laboratory for Bacterial Meningitis (NRLBM). The samples were collected from newborn infants with GBS disease within the first three months after birth.
Blinding	No blinding was performed in this study. Blinding was considered not to be necessary for our surveillance study as the nature of the study was observational and no intervention was given to the newborn infants. Therefore, there was no risk for bias by not blinding the investigators.

Reporting for specific materials, systems and methods

We require information from authors about some types of materials, experimental systems and methods used in many studies. Here, indicate whether each material, system or method listed is relevant to your study. If you are not sure if a list item applies to your research, read the appropriate section before selecting a response.

Materials & experimental systems

n/a	Involved in the study
☒	☐ Antibodies
☒	☐ Eukaryotic cell lines
☒	☐ Palaeontology and archaeology
☒	☐ Animals and other organisms
☐	☒ Human research participants
☒	☐ Clinical data
☒	☐ Dual use research of concern

Methods

n/a	Involved in the study
☒	☐ ChIP-seq
☒	☐ Flow cytometry
☒	☐ MRI-based neuroimaging

Human research participants

Policy information about studies involving human research participants

Population characteristics	Newborn with invasive GBS disease with the first three months from birth were included in the national surveillance of bacterial meningitis in the Netherlands. In this study we included 1338 infants who had invasive GBS disease between 1987 and 2016. Of these infants, 61.5% (823/1338) and 38.5% (515/1338) were from neonates with early onset disease (0 to 6 days, post-birth) and late onset disease (6 to 89 days, post-birth). By isolation source, 36.9% (494/1338) infants had a cerebrospinal fluid (CSF) sampled taken while bloodstream samples were collected from 63.1% (844/1338) infants. We did not have access to specific patient information, including gender.
Recruitment	The newborn with invasive GBS disease with the first three months from birth were included in the national surveillance of bacterial meningitis in the Netherlands. All isolates cultured from cerebrospinal fluid or blood from patients were submitted to the NRLBM at the Amsterdam UMC, University of Amsterdam, for further typing and storage, as part of the continuous surveillance of bacterial meningitis in the Netherlands. To avoid potential selection bias, we included randomly sampled isolates collected every year during the study period (1987-2016). For the present study patient data were anonymized. Additional institutional review board approval is not required for studying submitted strains with anonymised patient data.
Ethics oversight	For the present study patient data were anonymized. Additional institutional review board approval is not required for studying submitted strains with anonymised patient data.

Note that full information on the approval of the study protocol must also be provided in the manuscript.
