# Supplementary material for: Population genomics of Group B Streptococcus reveals the genetics of neonatal disease onset and meningeal invasion
Source: Nat Commun. 2022 Jul 21;13:4215. doi: 10.1038/s41467-022-31858-4 (PMC9304382; doi:10.1038/s41467-022-31858-4)
Supplement: Supplementary file 4 — Description of Additional Supplementary Files [file 41467_2022_31858_MOESM4_ESM.pdf]

Title: Supplementary Data 1:
Description: Summary of the Group B Streptococcus isolates analysed in this study.

Title: Supplementary Data 2:
Description: Number of sequenced GBS isolates per year stratified by serotype, disease onset, isolation tissue, and clonal complex (Related to Fig. 2).

Title: Supplementary Data 3:
Description: Relative frequency of GBS strains stratified by disease onset time and body isolation tissue (Related to Fig. 3).

Title: Supplementary Data 4:
Description: Summary of the SNPs and unitig sequences statistically associated with disease onset and meningeal invasion (Related to Fig. 4,5).

Title: Supplementary Data 5:
Description: Heritability estimates based on GEMMA, FaST-LMM, and GCTA methods (Related to Fig. 6).
